# Can Systematic Reviews Inform GMO Risk Assessment and Risk Management?

**DOI:** 10.3389/fbioe.2015.00113

**Published:** 2015-08-12

**Authors:** Christian Kohl, Geoff Frampton, Jeremy Sweet, Armin Spök, Neal Robert Haddaway, Ralf Wilhelm, Stefan Unger, Joachim Schiemann

**Affiliations:** ^1^Institute for Biosafety in Plant Biotechnology, Julius Kühn-Institut, Quedlinburg, Germany; ^2^Southampton Health Technology Assessments Centre (SHTAC), Faculty of Medicine, University of Southampton, Southampton, UK; ^3^Sweet Environmental Consultants, Cambridge, UK; ^4^Alpen-Adria Universität Klagenfurt-Wien Graz and IFZ-Inter-University Research Centre for Technology, Work and Culture, Graz, Austria; ^5^Mistra Council for Evidence-Based Environmental Management (EviEM), Royal Swedish Academy of Sciences, Stockholm, Sweden; ^6^Data Processing Group, Julius Kühn-Institut, Quedlinburg, Germany

**Keywords:** GMO, risk assessment, risk management, evidence synthesis, systematic review, bias

## Abstract

Systematic reviews represent powerful tools to identify, collect, synthesize, and evaluate primary research data on specific research questions in a highly standardized and reproducible manner. They enable the defensible synthesis of outcomes by increasing precision and minimizing bias whilst ensuring transparency of the methods used. This makes them especially valuable to inform evidence-based risk analysis and decision making in various topics and research disciplines. Although seen as a “gold standard” for synthesizing primary research data, systematic reviews are not without limitations as they are often cost, labor and time intensive and the utility of synthesis outcomes depends upon the availability of sufficient and robust primary research data. In this paper, we (1) consider the added value systematic reviews could provide when synthesizing primary research data on genetically modified organisms (GMO) and (2) critically assess the adequacy and feasibility of systematic review for collating and analyzing data on potential impacts of GMOs in order to better inform specific steps within GMO risk assessment and risk management. The regulatory framework of the EU is used as an example, although the issues we discuss are likely to be more widely applicable.

## Introduction

In many countries, genetically modified organisms (GMO) and their food or feed products have to undergo a stringent and science-based risk assessment before being placed on the market. In general, the risk assessment process follows a multi-step approach to identify and characterize a possible hazard and to determine the likelihood of its occurrence in order to conclude about a possible risk posed by a certain GMO. For each step, targeted scientific information has to be provided by the applicant who is in charge of applying for the release of a GMO into the environment to (1) frame the risk assessment and facilitate the elaboration and clarification of testable hypotheses and (2) allow risk assessors to provide scientific opinions on the overall safety in order to inform risk management. In the EU, for example, risk management includes specific monitoring activities and foresees the possibility for the evocation of safeguard clauses and emergency measures if new scientific information contesting a former risk conclusion becomes available (EC, 2001, 2003; EFSA, 2010a, 2011a). Data informing GMO risk assessment and risk management can take various forms and includes primary research data generated by the applicant and secondary research outcomes summarizing the available evidence base (EFSA, 2010a). The scientific literature assessing possible impacts of GMOs on human and animal health and the environment is sometimes characterized by heterogeneous results and conclusions (Devos et al., 2014b), compounded by the complexity and the diversity of test designs and the multitude of endpoints under investigation. In addition, the absence of tangible assessment criteria can hinder a clear and straightforward judgment about the validity and the relevance of the information for GMO risk assessment and risk management.

Systematic reviews are evidence synthesis approaches which have become well established in medical science to support evidence-based decision making (Guyatt, 1992). Their use is expanding to other disciplines to inform policy decisions, for example, in the areas of social welfare, international development, education, crime and justice^1^, environmental management^2^, and – more recently – food/feed safety assessment (EFSA, 2010b). Systematic reviews are based on a standardized and rigorous methodology to improve precision, minimize bias, and increase transparency, which are prerequisites for a robust synthesis of existing evidence. Thus, systematic reviews are especially valuable for synthesizing evidence relating to contentious topics for which stakeholders may hold differing views. Even though seen as a “gold standard” when summarizing primary data, systematic review methodology has limitations, for example it can be demanding on resources such as time, money, and manpower, and may not be worthwhile if the availability and robustness of primary research data (i.e., original data generated by one or more research studies) are limited. Thus, the decision to perform a systematic review should always consider both the potential limitations and benefits.

In this paper, we consider the added value systematic reviews can provide when summarizing primary research data and we consider the possible adequacy and feasibility of systematic review for collating and analyzing data on potential impacts of GMOs in order to inform specific steps within GMO risk assessment and risk management.

## Evidence Synthesis and Evidence-Based Decision Making

Evidence synthesis refers to the process of gathering together information to answer a question. This can be done in a number of different ways, depending upon the type of question to be answered and whether the answer is intended to be illustrative and approximate (e.g., identifying general patterns or trends) or quantitatively reliable and precise (e.g., determining structural or input parameters for a quantitative model). A commonly used approach for answering scientific questions of both types is to conduct a literature review. Reviews of the literature vary considerably in how they are conducted and if they do not follow an *a priori* defined and documented procedure that employs explicit means to identify, critically appraise, and evaluate included studies they are usually referred to as “traditional” or “narrative” reviews.

### What is a systematic review and why conduct one?

A systematic review is a structured, reproducible, and rigorous approach for answering a specific question (EFSA, 2010b). The key advantages of a systematic review over other types of evidence synthesis are that a systematic review can answer a question in a transparent manner that minimizes bias and maximizes precision. Bias is minimized by following a standardized procedure, comprising eight steps, as illustrated in Table 1.

**Table 1:** **Core steps in the conduct of a systematic review**.

Steps of the systematic review	Key procedures
1. Preparing the review	The review question is clearly specified and a protocol detailing the review methods is developed. The protocol should be subject to peer review and could include stakeholder involvement in its development or peer review
2. Searching for evidence	An extensive search is conducted based on a pre-specified search strategy which aims to identify all relevant evidence, reducing the risk of selection bias
3. Selecting studies for inclusion or exclusion in the review	The identified evidence is assessed against eligibility criteria specified in the protocol to ensure that only appropriate evidence is included in the review, reducing risk of bias from selective evidence inclusion
4. Collecting data from the included studies and creating evidence tables	Data are collected from the included studies using a standard, pilot-tested form to ensure that only relevant data are extracted, in a way that minimizes errors
5. Assessing methodological rigor of included studies	The primary research studies are critically assessed for study rigor, in particular any methodological aspects that could lead to risk of bias (referred to as internal validity) or issues of generalizability (referred to as external validity)
6. Synthesizing data from included studies, possibly including meta-analysis	Pooling of quantitative outcomes across similar primary studies may be conducted to improve precision of the answer, subject to the studies meeting adequate pre-specified standards of rigor
7. Presenting data and results	Presentation of results is transparent, including a clear specification of the reasons why studies were excluded from the review and clear specification of how the analysis was conducted, including how any studies at risk of bias were handled
8. Interpreting results and drawing conclusions	The interpretation of qualitative and/or quantitative results takes into account any limitations of the included primary studies as well as any limitations of the review process. Stakeholders could be involved, e.g., if the draft systematic review report is circulated among stakeholders for comment. Implications for research/policy/practice are provided but reviews should ensure that these do not over-reach the review findings

#### The Importance of Bias

Bias is defined as a systematic deviation in study results from their true value, i.e., it means either an underestimation or overestimation of the true value. Bias should not be confused with statistical uncertainty as a result of random error, which is present in all research studies. Random error reflects uncertainty in the study result due to statistical limitations of the study design and, as the name implies, it reflects inaccuracy of estimation that is distributed randomly around the true result. Often, random error can be reduced by increasing the sample size in a research study, or by quantitatively combining the results of similar studies in a meta-analysis (subject to the studies being adequately comparable), hence improving the precision of the result (Glass, 1976). Bias, on the other hand, refers to a systematic error which cannot be reduced by increasing the sample size or pooling study results in a meta-analysis. If bias is present in primary research studies, their results are likely to be incorrect. Traditional non-systematic reviews of evidence which do not formally assess the rigor of primary research studies would not be able to detect this.

Bias in research studies can arise for a variety of reasons. Poor design of a research study may mean that it consistently underestimates or overestimates the true value of an outcome and the study researchers may not be aware of this. In some cases, researchers may have a vested interest in a particular outcome and this could lead, either intentionally or unintentionally, to various types of bias. Considerable experience from evidence synthesis in health research has shown that where bias is present it often leads to overestimation of beneficial outcomes, e.g., exaggerating the actual benefits of an intervention such as a drug treatment (Higgins and Green, 2011).

A number of tools are available for assessing the risk of bias in primary research studies but these depend on the study design and are mainly developed for randomized studies, e.g., in health research (Higgins and Green, 2011), and in research involving laboratory animals (Hooijmans et al., 2014). Even if a specific tool is not available to guide a risk of bias assessment for study designs relevant to a particular question, likely sources of bias have to be considered and a critical appraisal strategy has to be pre-specified in the review protocol.

In a traditional narrative review, bias could arise either from the primary studies included in the review or from the evidence synthesis process itself, for example if reviewers are selective in the evidence that they include or in the analysis methods they use. This latter bias in the review process itself is mitigated in systematic reviews through transparent and objective conduct and reporting of the processes undertaken.

#### Principles of Critical Appraisal in Evidence Synthesis

The critical appraisal of primary research studies is often referred to as “quality assessment” but the term “quality” is rather vague, without a consistent meaning, and has been interpreted by some as being possibly offensive to study investigators (Higgins and Green, 2011). When conducting a critical appraisal of primary research, it is important that the assessment focuses on aspects of methodological rigor that will have a direct bearing on interpreting the results of the evidence synthesis. There are two such aspects that need to be considered. These are the risk of bias and the generalizability of the findings. Studies which are conducted in such a way that they are considered to be at low risk of bias are said to have high “internal validity” and studies whose results are directly generalizable to answer the review question are said to have high “external validity.” These two aspects of critical appraisal are fundamentally important but differ in the way they are handled. Whilst the internal validity of a study (i.e., the extent to which it is likely to suffer from bias) is a property of the primary study in question, the external validity of a study is not a property of the study itself but is related to the question being answered (e.g., results of a well-conducted study on a GM crop might be generalizable to some countries but not others – so external validity of the findings would depend on which country a risk assessment question refers to).

Detailed consideration of critical appraisal and tools to aid in undertaking critical appraisal assessments in systematic reviews have been published in the medical science literature and, more recently, in environmental sciences. These considerations cover different types of biases, study designs, and the appropriateness of assessment tools (e.g., medical research: Katrak et al., 2004; meta-analyses in agronomy: Philibert et al., 2012; environmental research: Bilotta et al., 2014).

#### What Makes Systematic Reviews Different?

Systematic reviews achieve the objectives of minimizing bias, maximizing precision, and ensuring transparency and reproducibility in a number of ways. Systematic reviews are best suited to answer specific questions. In general, if a primary research study can be envisaged that could answer a question, then it is likely that the same question can be addressed by a systematic review. A useful concept for considering whether a question would be answerable by a systematic review is to analyze the question structure in terms of “key elements” (EFSA, 2010b; Aiassa et al., 2015). In questions about interventions, the key elements are the population(s) (P), intervention(s) (I), comparator(s) (C), and outcome(s) (O), all of which must be specified for the question to be answerable by a systematic review. A systematic review is based on a pre-specified protocol which ensures that the overall evidence synthesis is objective and should not be influenced by selective use of evidence or methods that could introduce errors or bias. The protocol should, ideally, be peer reviewed and updated if necessary before a review starts (step 1, see Table 1). The protocol should specify how each of the steps of the review will be conducted and by whom. Searching (step 2) aims to identify all relevant evidence using a pilot-tested search strategy and a range of evidence sources, including gray as well as academic literature. This is to reduce the risk of publication bias (i.e., the selective identification of the evidence due to positive or negative results being published preferentially over no-effect results in more accessible literature sources). The process for including relevant evidence into a systematic review (step 3) is based on clear selection criteria specified *a priori* in the protocol to ensure that the selection process is as objective and impartial as possible. Collection of data from the included studies (step 4) is also based on pre-specified criteria to ensure that the data collected directly inform the analysis. Data extraction forms are usually included in a systematic review report (e.g., in an appendix) so that the relationship between the data which are collected and those which are analyzed is transparent, minimizing the risk of unplanned selection of data subsets for preferential analysis. A systematic review always includes a critical appraisal step where the methodological rigor and generalizability of the included primary studies is evaluated (step 5). Whilst internal validity should always be assessed in a systematic review, the assessment of external validity may or may not be necessary depending upon the nature of the review question and the primary studies that may answer it. Studies which are considered to be at high risk of bias may then be either excluded from the data synthesis (step 6) or included in sensitivity analyses to clarify their impact on the review’s conclusions. A systematic review may or may not support a quantitative pooling of outcomes across studies, i.e., a meta-analysis. This depends, among other factors, on whether the studies are methodologically and statistically homogeneous. The reasons for conducting or not conducting a meta-analysis should be transparently stated and consistent with the planned approach specified in the review protocol. The presentation of data and results (step 7) should follow a clear and logical structure so that the roles of the primary studies informing the review’s results can be readily deduced and reasons for the exclusion from analysis of any studies which met the initial inclusion criteria are explained. The final part of a systematic review, where the results are interpreted and conclusions drawn (step 8), should demonstrate that the conclusions are based directly on the results of the review, and should also include a critical reflection stating any limitations of the review itself and the implications they have for the review’s conclusions.

A comparison of systematic against traditional narrative reviews is shown in Table 2.

**Table 2:** **Comparison of key aspects of traditional reviews and systematic reviews**.

	Traditional “narrative” reviews	Systematic reviews	Reasons why systematic reviews may be advantageous for synthesizing evidence compared to non-systematic traditional (narrative) reviews
Review question	Often broad in scope	Focused and explicit	The question is focused and a systematic review directly answers it, based on evidence identified explicitly as being the most relevant and robust
Criteria for inclusion or exclusion of studies	Not always explicitly stated	Pre-defined and documented; applied in a verifiable manner	The scope of the evidence is explicitly clear, meaning that evidence cannot be gathered selectively (systematic reviews reduce bias), irrelevant evidence is avoided (systematic reviews ensure efficiency), criteria are pre-defined (systematic reviews enable stakeholder involvement), and the criteria and process aim to be objective (systematic reviews reduce ambiguity or subjectivity of interpretation)
Review method	Seldom reported	Reported and also pre-defined in a protocol	By explicitly and transparently reporting how and why evidence is collected, the synthesis can be clearly defensible, reproducible, and may be readily updated. Being a systematic and standard approach, the robustness of systematic reviews can be easily checked
Literature search	Not always extensive	Structured to identify as many relevant studies as possible	All relevant evidence is considered (systematic reviews identify and/or minimize publication bias) or, in cases where evidence is not included (e.g., confidential data) this can be made explicit so as to fully inform interpretation
Methodological critical appraisal of included studies	Variable	Included, typically using a critical appraisal tool	Critical appraisal of the included evidence can ensure that systematic review findings reflect the truth in terms of their magnitude and direction (i.e., bias is minimized) with an appropriate degree of certainty – i.e., the estimates of outcomes and their precision levels are both valid. This is an important ‘filter’ in evidence synthesis that enables less rigorous evidence to be identified and handled appropriately
Critical appraisal example: reporting of study outcomes	Selective reporting; often of study author’s interpretation	Full reporting of relevant outcomes (numerical results)	By exposing and/or controlling for selective reporting, systematic reviews can minimize reporting bias which could be a problem in cases where stakeholders have vested interests in certain outcomes
Synthesis	Usually narrative, sometimes selective	Quantitative synthesis (meta-analysis) when possible	Where possible, systematic reviews make use of the best available evidence to improve precision of the answer; explicit exploration of assumptions and limitations is possible, e.g., in sensitivity analyses

#### Systematic Reviews Facilitate Stakeholder Involvement

A systematic review has the potential to minimize bias by encouraging researchers to find and transparently document all relevant evidence. Stakeholders have an important role to play, e.g., in helping to determine which questions in a risk assessment have highest priority for a systematic review. They may also provide guidance to inform the review processes. Although in theory relevant stakeholders could contribute to any type of evidence synthesis, including traditional narrative reviews, the structured systematic approach of systematic reviews is particularly well-suited for involving stakeholders. It is important to ensure that stakeholder participation is appropriately balanced rather than representing only certain viewpoints and neglecting others.

For a systematic review to function efficiently, it is generally not feasible to involve stakeholders in all the steps, particularly if the range of stakeholders is very broad. But there are key points in the review process where stakeholders could, and arguably should, be involved. These are in preparing the review (step 1) and interpreting the results and drawing conclusions (step 8). A relatively straightforward approach for involving stakeholders in preparing the review is to invite them to comment on or contribute to developing the review protocol. This could be done as part of the review planning and/or formal peer review of the protocol. A relatively straightforward approach for involving stakeholders at the point of interpreting the results and drawing conclusions could be to invite them to comment on a draft of the systematic review report, and/or to be involved in the formal peer review of the final published version of the report. If stakeholders do contribute to a systematic review, it is important that their roles and interests are clearly acknowledged (Saan et al., 2015). Another option is to recruit stakeholders to an advisory group which can inform the review, but to avoid an unbalanced influence of specific stakeholders the role of the advisory group should be clearly specified in the review protocol and subsequently adhered to.

When involving stakeholders in an evidence synthesis, it is important that the stakeholders are clearly informed of the purpose of the evidence synthesis so that they can comment in an appropriate manner.

### Limitations of systematic reviews

Whilst systematic reviews offer valuable opportunities to reliably synthesize evidence on specific topics, they are not without limitations. Systematic reviews should be based on a specific, well-defined review question that is established at the start of the review. This may prove to be a challenge, particularly where topics are dynamic and the precise area of stakeholder interest may be fluid, but it is vital to ensure that the review remains on target and the outputs are useful. Furthermore, as a systematic review by definition has a minimum number of steps which should be completed by a minimum number of people (usually a review team is recruited), it can be relatively resource intensive compared to a traditional narrative review and cannot provide an immediate answer to a question (since the development of the review protocol and then following the subsequent steps of the process usually take months rather than days or weeks). However, the relatively high resource requirement and lack of immediate results from a systematic review have to be weighed against the need for an answer that is valid and precise. As the validity and the precision of synthesis outcomes depend on the reliability and the quantity of included studies, performance of systematic reviews may only be useful to support regulatory decision-making processes when sufficient and robust primary data are available.

### Systematic reviews can inform evidence-based decision-making processes

An example where systematic reviews are employed routinely as a standard approach for evidence synthesis in support of regulatory decision making is the technology appraisal process used by the National Institute for Health and Care Excellence (NICE) for approving the use of health technologies (including drugs, other treatments, devices, and tests) in England and Wales (NICE, 2013). The NICE appraisal process requires that applicants seeking approval of a health technology should provide systematic review(s) of the clinical effectiveness of the technology. The process is highly structured and involves stakeholders (including independent academic groups, companies and sponsors, healthcare professionals, commissioners of health services, and patient or carer representatives) at several steps, including the initial definition of the scope of the technology appraisal. The evidence submitted by an applicant, including its systematic review(s), is critically assessed by an independent academic assessment group which reports to NICE, and further information or analyses may be requested by NICE from the applicant if necessary. A final decision on the approval of the technology is made at one or more appraisal committee meetings which include NICE, the applicant, the independent assessment group, and other stakeholders. The appraisal process and the rationale for the final decision are reported transparently on the NICE website for each technology that is assessed^3^. The use of systematic reviews in the NICE decision-making process is well established: between 1st March 2000 and 31st March 2015, NICE conducted 553 technology appraisals which yielded 578 individual recommendations^4^. Even though the approval process followed by NICE differs in some ways from the regulatory approval process of GMOs, it illustrates the value that systematic reviews can have in supporting regulatory decision making.

## GMO Risk Assessment and Risk Management Require the Provision of Targeted Scientific Information

While regulation of GMOs differs between jurisdictions, the decision-making process is always based on science-based risk assessment. The underlying frameworks aim to identify, characterize, and evaluate the likelihood that an adverse effect might occur and to determine the need for the implementation of risk management measures (EPA, 1998; EC, 2001, 2003; Environment Canada, 2012).

Environmental risk assessment of GMOs in the EU follows the EFSA guidance for genetically modified plants (EFSA, 2010a) and generally comprises six major steps, including (1) problem formulation and hazard identification, (2) hazard characterization, (3) exposure characterization, (4) overall risk characterization by placing the magnitude of consequences in relation to the probability of their occurrence, (5) the development of risk management strategies, and (6) an overall risk evaluation (EFSA, 2010a). Risk assessment of GMOs for food or feed safety evaluation follows a similar approach (EFSA, 2011a). The core of each risk assessment is built up by a comparative safety assessment which considers the characteristics of the GMO and its closest non-GM counterpart in order to identify possible hazards that further determine the scope of the risk assessment (Kok and Kuiper, 2003). Furthermore, EU regulation demands, as part of the risk management, the development of a post-market environmental monitoring plan in order to “identify any direct or indirect, immediate and/or delayed adverse effects of GMOs, their products and their management to human health or the environment, after the GMO has been placed on the market” (EFSA, 2010a). In addition, EU regulation foresees the possibility for the evocation of safeguard clauses and emergency measures if new scientific information contesting former risk assessment conclusions becomes available (EC, 2001, 2003).

In order to draw conclusions about possible risks, targeted scientific information is considered for the different risk assessment/risk management steps, so that each step supplies sufficient evidence in order to quantify and describe each risk identified. The evidence base to satisfy the respective data requirements may derive from a variety of sources, such as primary and secondary research studies or, in cases lacking primary evidence, further research, e.g., data generated by the applicant. In line with the principle of the comparative safety assessment, the most frequent questions likely to arise in GMO risk assessment and risk management would have a PICO structure. In such cases, the *population* is representing the entity under investigation (e.g., organisms exposed to the GMO), the *intervention* is usually the GM plant, trait, or event that the population is exposed to, the *comparator* is normally the closely related (e.g., near-isogenic) non-GM organism providing the baseline characteristics the intervention should be compared to, and the *outcome* is specifying the assessment/measurement endpoints being considered. Questions about occurrence or consumption, or about the accuracy of test methods, may also be relevant for a complete impact assessment, and these have different key elements (i.e., other than PICO structure) which would need to be specified [for more detail see EFSA (2010b), Aiassa et al. (2015)].

Thus, the systematic review methodology might offer a standardized approach to provide robust data compatible with the demands of GMO risk assessment and risk management. To be clear, systematic reviews might be considered as a robust means of collating the evidence which is used to inform the different stages of the assessment process but should not be seen as an integral part of GMO risk assessment or risk management. In the following sections, we discuss how systematic reviews could provide rigorous syntheses of GMO data, such as comparative impact data, and consider the benefits, challenges, and limitations of performing systematic reviews for this purpose (see Table 3).

**Table 3:** **Systematic reviews and their adequacy to inform GMO risk assessment and risk management**.

Steps in GMO risk assessment and risk management according to EFSA (EFSA, 2010a) and Directive 2001/18/EC (EC, 2001)	Data typically informing each step	Added value of SR methodology	Challenges/limitations for SR performance
**GMO RISK ASSESSMENT**			
**Problem formulation/Hazard identification (Devos et al., 2012)**	Crop/trait/event and related management systems	*Potential added value when synthesizing primary research outcomes from sources 1 and 2*	Sufficient primary research data would have to be available for a SR to be worthwhile and add value, which is not likely for novel traits/events or rarely used events Could be time/labor/cost intensive depending upon the size of the evidence base Question prioritization (based on problem formulation and/or conceptual models) might be needed
Identification of characteristics of the GMO that might cause potential adverse effects Identification of exposure pathways Definition of assessment endpoints Formulation of testable hypotheses to frame subsequent RA steps Elaboration of analysis plans	*Sources* (1) Published scientific literature (2) Unpublished scientific studies (3) Topic expert opinions	Increase precision Minimize bias Ensure transparency Facilitate stakeholder involvement Clarify uncertainty	
		*Resulting benefits*	
		SR could support a rigorous evaluation of relevant parameters, e.g., assessment endpoints and exposure pathways (if sufficient literature reporting relevant parameters exists, the reliability of the parameter estimates could be assessed and precision of the final estimate employed in the RA, improved by quantitative pooling where appropriate) SR could provide defensible answers to support decisions when framing the scope of subsequent RA steps SR could support targeted communication between assessors (and other stakeholders), by providing information in a standardized, well-structured way	
		*SR and topic expert opinions (source 3):*	
		SR do not generally synthesize expert opinions; however, expert opinions are often valuable in setting the review question and developing the protocol and might contribute to decisions regarding the prioritization of different review questions	

**Hazard/exposure characterization (EFSA, 2010a, 2011a)**	Event/trait and related management systems	*Potential added value when synthesizing primary research outcomes from sources 1 and 2*	Sufficient primary research data would have to be available for a SR to be worthwhile and add value, which is not likely for novel traits/events or rarely used events Could be time/labor/cost intensive depending upon the size of the evidence base Question prioritization might be needed
Qualitative and/or quantitative evaluation of potential adverse effects Quantitative estimation of the likelihood of exposure	*Sources* (1) Published scientific literature (2) Unpublished scientific studies	Increase precision Minimize bias Ensure transparency Facilitate stakeholder involvement Clarify uncertainty	
		*Resulting benefits*	
		SR (including meta-analysis) could provide valid quantitative estimates with known precision regarding the intensity or likelihood of a hazard SR could support a targeted communication between assessors (and other stakeholders), by providing information in a standardized, well-structured way	

**Risk characterization (EFSA, 2010a, 2011a)**	Event/trait and related management systems	SR would be unlikely to add value here, since this step determines and quantifies risks based on data collected, analyzed, and interpreted at the previous steps	Not applicable
Qualitative and/or (semi-) quantitative estimation, including attendant uncertainties of the probability of occurrence and severity of known or potential adverse effects	*Sources* Information gathered during hazard/exposure characterization		

**Development of risk management strategies (EFSA, 2010a)**	Crop/trait/event and related management systems	*Potential added value when synthesizing primary research outcomes from sources 1 and 2*	Could be time/labor/cost intensive depending upon the size of the evidence base Primary data have to be available and accessible Question prioritization might be needed
Reduce the identified risks to a level of no concern and consider defined areas of uncertainty When possible the reduction of risk should be quantified The reliability and efficiency of risk management characteristics should be assessed	*Sources* (1) Published scientific literature (2) Unpublished scientific studies	Increase precision Minimize bias Ensure transparency Facilitate stakeholder involvement Clarify uncertainty	
		*Resulting benefits*	
		SR may support a rigorous evaluation of risk management measures and strategies SR could support a targeted communication on assessment details between assessors (and other stakeholders), by providing information in a standardized, well-structured way	

**Overall risk evaluation and conclusions (EFSA, 2010a)**	Event and related management systems	SR would be unlikely to add value here, since this step draws conclusions about an overall risk posed by a GMO, based on data collected, analyzed, and interpreted at the previous steps	Not applicable
Evaluation of the overall risk	*Sources* Information gathered during previous RA steps		

**GMO RISK MANAGEMENT**			
**Monitoring/general surveillance (Devos et al., 2012)**	Trait/event and related management systems	*Potential added value when synthesizing primary research outcomes from sources 1, 2, and 3*	Updating an existing SR might be time/labor/cost intensive if a large amount of new information has to be included If a SR addressing a specific question does not already exist conducting a full new SR could be time/labor/cost intensive Primary data have to be available and accessible Question prioritization might be needed
Case-specific monitoring**:** Targeted investigations to reduce uncertainties identified in previous steps General surveillance: tracking system states after market release of a GMO to anticipate cumulative and unintended effects	*Sources* (1) Scientific literature (2) Unpublished scientific studies (3) Farm questionnaires (4) Existing monitoring networks	Increase precision Minimize bias Ensure transparency Facilitate stakeholder involvement Ensure updatability Clarify uncertainty	
		*Resulting benefits*	*SR and existing monitoring networks (source 4)*
		SR (including meta-analysis) could provide valid quantitative (or qualitative) estimates of relevant outcomes with known precision A SR could facilitate integration and weighing of new studies by providing a consistent and transparent evaluation scheme that is readily updatable	The external applicability of information from existing monitoring networks might be questionable; if so this would have limited value for GMO RA

**Safeguard clause/“emergency measures”**	Event	*Potential added value when synthesizing primary research outcomes from sources 1 and 2*	Depending on the amount of new information, a timely answer might not be possible Primary data have to be available and accessible
Determine the need for RA update based on new available evidence	*Sources* (1) Scientific literature (2) Unpublished scientific studies	Increase precision Minimize bias Ensure transparency Ensure updatability Clarify uncertainty	
		*Resulting benefits if new data are within the scope of an existing SR relevant to the RA*	
		Weighing of the information and assessment of its impact on previous risk/safety conclusions (e.g., via sensitivity analysis) Communication about possible shortcomings affecting the reliability of the new information, facilitated by SR since relevant information is provided in a structured, standardized way, including objective critical appraisal	

*This table illustrates the different risk assessment (RA) and risk management (RM) steps which use scientific research data in order to conclude about the safety of a genetically modified organism (GMO). It identifies the nature of the considered data, the possible added value provided by systematic reviews (SR), and depicts the challenges and limits for their conduct*.

### Are systematic reviews appropriate tools to inform specific GMO risk assessment/risk management steps?

To help in weighing up the appropriateness of systematic reviews to inform GMO risk assessment and risk management, potential benefits, challenges, and limitations relevant for each step are briefly summarized here.

Potential benefits to risk assessment and risk management of systematic reviews are as follows:

- Increasing precision by means of a quantitative data synthesis, e.g., via meta-analysis, thereby facilitating the clarification of uncertainties.
- Minimizing bias by the elaboration of a review protocol and by the impartial application of assessment criteria.
- Increasing transparency by assuring thorough documentation of the review process.
- Facilitating stakeholder involvement (e.g., by discussion of the review protocol).
- Facilitating updating by following a standardized and thoroughly recorded procedure.
- Facilitating a transparent communication of assessment details by means of the review report might increase the traceability of review conclusions for risk assessors and risk managers (e.g., why were certain studies included in the review and others not, which criteria were applied during critical appraisal, and how were the appraisal results considered during the synthesis step?).

Potential challenges and limitations of systematic reviews are as follows:

- Systematic reviews can be resource intensive and are thus not always feasible.
- Systematic reviews do not provide an immediate answer to a question.
- Where answers are required for many questions, prioritization of questions may be appropriate. One prioritization approach suggested by Aiassa et al. (2015) would consider how influential the answer to the question will be for the overall risk assessment, with those questions (or model parameters) which have the greatest influence being prioritized for evidence synthesis.
- Sufficient primary research data would have to be available for a systematic review to usefully inform risk assessment or risk management; this may not be likely for novel or rarely studied traits or events (it is more likely that there would be sufficient evidence for stacked events, where the respective single events have already been studied in detail, or for renewals of approval applications).

Due to these possible limitations, the appropriateness of systematic reviews might strongly depend on the specific topic and question under assessment and a decision for or against their performance would have to be made on a case-by-case basis. Possible points where systematic reviews could inform the specific risk assessment and risk management steps are considered in the following sections, and are illustrated, with their potential strengths and limitations, in Table 3.

#### Can Systematic Reviews Inform GMO Risk Assessment?

Each risk assessment begins with the identification and formulation of a problem in order to identify the areas of greatest uncertainty or concern to be considered during risk characterization (EPA, 1998; Hill and Sendashonga, 2003; EFSA, 2010a; Devos et al., 2012).

Central steps at the problem formulation stage are the definition of assessment endpoints, which are explicit and unambiguous targets for protection extracted from legislation and public policy goals, and the identification of possible hazard(s) and exposure route(s) through which the GM plant may adversely affect or interact with the environment (EPA, 2003; Sanvido et al., 2012). These enable testable hypotheses to be derived to support a quantitative evaluation during hazard and exposure characterization (EFSA, 2010a). Depending on the scope of the different risk assessment models, relevant information facilitating their development and the elaboration of a final risk analysis plan could be crop, trait, or event specific and may stem from various sources, including scientific literature, topic expert opinions, and new research data e.g., generated in the context of applications (i.e., unpublished scientific studies).

Systematic reviews could contribute to problem formulation (Table 3). Expert knowledge informs the development of the problem formulation and hence the identification of potential review questions and the elaboration of review protocols. However, various different questions being supported by a different amount of available evidence could arise during problem formulation and it would not be feasible (and may not be considered necessary) to answer all of these with systematic reviews. This implies that a prioritization process could be helpful to clarify where robust synthesis of the evidence could be most important and worthwhile. Problem formulation is typically supported by a conceptual model which sets out the protection goals, assessment endpoints, and measurement endpoints of the risk assessment and risk management (Wolt et al., 2010; Gray, 2012). The conceptual model could provide a logical structure for identifying which key variables and pathways (e.g., stressors, receptors, and exposure routes) systematic review might be applied in order to answer questions about them generated in the problem formulation.

Based on problem formulation outcomes, systematic reviews might be applied to synthesize evidence to be considered during hazard and exposure characterization and during the development of risk management strategies (see Table 3). In both cases, the considered evidence may stem from the published scientific literature and/or from unpublished scientific studies.

During hazard and exposure characterization, potential adverse effects (hazards) are characterized by providing (1) a quantitative and/or qualitative estimate of the nature of the associated harm and (2) a quantitative estimation of the exposure and frequency or likelihood of the hazard (EFSA, 2010a). For example, trait- and event-specific information could be provided by a systematic review assessing impacts of Bt-maize cultivation on the abundance or ecological function of non-target organisms (Meissle et al., 2014).

In the course of the development of risk management strategies, questions about whether a characterized risk can be sufficiently managed to meet acceptable levels of concern, and about the reliability and efficiency of the proposed risk management strategies will be addressed (EFSA, 2010a). Here, systematic reviews could provide robust statements about factors which can influence the efficiency of management strategies. For example, the information provided by a systematic review can inform the baseline susceptibility assessment of different lepidopteran and coleopteran maize pests to Bt-proteins (Gathmann and Priesnitz, 2014).

#### Can Systematic Reviews Inform GMO Risk Management?

##### Monitoring and general surveillance

After approval for the commercial release (cultivation and/or import and processing) of a GMO in the EU, it is mandatory that it is monitored for the occurrence of adverse effects (EC, 2001; EFSA, 2010a, 2011a).

Monitoring plans should consider case-specific monitoring aimed at assessing risks and/or uncertainties identified during the risk assessment process. In addition, post-market environmental monitoring also includes a requirement for general surveillance to be performed to assess any unanticipated effects arising from the use of the GMO.

Data informing the general surveillance can stem from a multitude of different sources in heterogeneous formats (Graef et al., 2008; Wilhelm et al., 2009; Smit et al., 2012). Information sources may include scientific literature, data generated by the applicant through farm questionnaires, or data from existing monitoring networks (EFSA, 2011b). Once a considerable evidence base is available, the updatability of systematic reviews could allow the integration and weighing of new studies by following established protocols. This would further support a targeted discussion about (new) evidence arising from monitoring data and inform risk assessors, managers, or other stakeholders of any changes in review outcomes caused by their inclusion.

In general surveillance, there is an obligation for applicants to review existing data relating to their event. This will therefore require reviewing event as well as related trait data. At present, there is a recommendation to follow the systematic literature review methodology to select relevant evidence (EFSA, 2011b) in order to increase the defensibility of the information provided. The scope of the general surveillance, namely the detection of any unintended effect that was not anticipated in the risk assessment, would be much too broad for a single systematic review. Thus, specific questions would need to be identified, prioritized, and (depending on the availability of resources) subjected to one or more systematic review(s).

As mentioned above, further information to be considered during general surveillance may stem from farm questionnaires and existing monitoring networks (or monitoring reports). Farm questionnaires frequently provide categorized qualitative data (Berensmeier et al., 2006) and thus a systematic review might in principle be applied to assess the impact of GM crop cultivation on enquired variables over time at different integration levels (trait or event). The integration of information provided by existing monitoring networks for general surveillance is largely hindered by a poor comparability between the data formats (e.g., inconsistencies in recording and sampling methods) (EFSA, 2011b; Smets et al., 2014). By providing a standardized approach for assimilating evidence, systematic reviews might contribute to improved harmonization of the data collection.

In case-specific monitoring, applicants are asked to discuss the results of the monitoring in relation to current knowledge in order to clarify uncertainties identified during the risk assessment (EFSA, 2010a, 2011b). This could be done by a systematic review by providing a robust summary of the available evidence base for a specific question.

##### Safeguard clauses and emergency measures

Once a GMO is approved, Directive 2001/18/EC and Regulation (EC) 1829/2003 allow member states to invoke safeguard clauses or further emergency measures to restrict or prohibit the marketing of the GMO on their territory if new information relevant to the safety of the GMO becomes available. Once the EC is notified by a Member State about such a request, EFSA receives the mandate to evaluate the scientific justification for such an invocation based on the information submitted by the Member State (EC, 2002; Devos et al., 2014a). The possibility to invoke such measures is not likely to be affected by the recent opt-out provision, allowing EU member states to restrict or ban the cultivation of GM crops on their own territory without following a science-based reasoning (EC, 2015).

Data supporting the invocation of safeguard clauses and emergency measures may be derived from scientific literature or any sources that provide relevant primary research findings. In order to decide and to communicate explicitly if the new information overturns former risk assessment conclusions and risk management decisions, a systematic review would be a tool of choice to scientifically determine whether the addition of the new information changes the outcome of a systematic review, provided that the new data are within the scope of an existing systematic review already used to inform GMO risk assessment and are available in the public domain. In this case, a systematic review might be updated to determine the weight and impact of the new information on the overall conclusions e.g., via sensitivity analysis.

However, updating and reanalyzing a systematic review may be too time consuming, hindering the provision of a timely answer which may be required if there are concerns about imminent harm. In such a case, the critical appraisal criteria made explicit in the protocol could be directly applied to assess the robustness of the new information but a statement regarding their possible impact on former review conclusions could only be given once updating is finalized.

## Discussion

By increasing precision and transparency, minimizing bias, facilitating stakeholder involvement, and clarifying uncertainty, systematic reviews can provide robust quantitative and/or qualitative answers to specific scientific questions and are frequently applied e.g., in medical sciences to support decision-making processes (see Systematic Reviews Can Inform Evidence-Based Decision-Making Processes). Besides an increase in the robustness of evidence synthesis outcomes, an additional advantage of systematic review methodology might stem from a structured and transparent presentation of assessment details by means of the review protocol. Thus, the scientific basis for any decision to be made during the synthesis process is made explicit *a priori*, thereby increasing the traceability of review conclusions for risk assessors and risk managers. Systematic review performance is not free from challenges and limitations as it may be highly time, labor, and cost intensive. In addition, sufficient primary data have to be available for a systematic review to be worthwhile. Thus, when adopting systematic review methodologies for the support of decision-making processes, three fundamental questions would need to be considered. (1) Would one or more systematic reviews provide data compatible with the demands of the decision-making process? (2) Would the performance of these systematic reviews be feasible when considering the associated challenges and limitations? (3) Would the review(s) add value to the decision-making process compared to existing methods used for synthesizing the evidence?

Genetically modified organisms risk assessment and risk management require comparative quantitative and/or qualitative estimates of impacts in order to draw conclusions about the risks of a GMO. PICO-type questions already resemble the concept of the comparative assessment, supporting the appropriateness of systematic reviews to inform specific risk assessment or risk management steps about possible impacts of a GMO on the environment and on human and animal health. As most of the information to be provided in the approval process for GMOs is focused on a specific event, the availability of primary research data could be a major limitation restricting the use of systematic reviews. Thus, systematic reviews might only be feasible on a case-by-case basis where the available evidence base would justify their conduct. By contrast, in the EU the Implementing Regulation (EU) No 503/2013 (EC, 2013) on applications for authorization of genetically modified food and feed requests the applicant to “ include a systematic review [….] on potential effects on human and animal health of the genetically modified food and feed covered by the application”. In practice, this would not be feasible with a single systematic review, which requires a specific question to be specified, so the problem would need to be broken down into a set of more specific questions. Hence, the intended meaning of this statutory requirement needs clarification.

A considerable challenge for systematic reviews is the problem of publication bias. This refers to the tendency for scientific journals to publish papers that report significant or “novel” results, whereas studies that do not reveal significant effects may not even be submitted for publication. This has been well documented and investigated in the medical sciences (Parekh-Bhurke et al., 2011) and although likely also in the field of GMO risk assessment, so far no research has quantified the extent of publication bias in relation to GMO research. A central tenet of systematic review methods is that the possibility of publication bias should be minimized by undertaking searches for gray literature, which includes ‘file-drawer’ studies, such as theses and pre-prints, non-standard academic reports, such as meeting abstracts, and practitioner-generated research, such as organizational reports and evaluations (Haddaway and Bayliss, 2015). Statistical methods are available to assess publication bias (Higgins and Green, 2011; Jin et al., 2015). However, a considerable number of studies in the field of GMO impact assessment have been conducted for regulatory purposes and have never been published. Such studies are typically included in applications for market releases and can in many jurisdictions only be accessed by regulatory bodies. Hence, their identification and inclusion in publicly accessible systematic reviews would face considerable hurdles. For example, in the EU, first relevant studies need to be identified by gaining read access to an application (from EFSA), and then permission for the further use of study data must be obtained from the data owner. Therefore, a considerable body of evidence would likely be excluded from evidence syntheses that require data from regulatory applications.

In principle, a systematic review could be a robust way to assess new scientific evidence since it can transparently describe the weight and impact of the new information on existing review outcomes and conclusions. However, if a timely answer is needed e.g., in the case of urgent policy questions, it may only be feasible to systematically assess the new information if it is within the scope of an existing systematic review. If no systematic review with the same scope is available, it is unclear what to do in such a situation, e.g., whether it may be acceptable to limit the searches to specific sources of information to speed up the review process. Although the notion of a “rapid review” has emerged, there is currently no consistent definition of one (Harker and Kleijnen, 2012). Where the full systematic review process including development and peer review of the protocol would not be feasible in the short timescale required for some questions to be answered, a pragmatic review approach would have to be agreed and the limitations of such an approach should be clearly stated.

A further challenge likely to arise concerning the use of systematic reviews in risk assessment and risk management is how to decide which of the many questions arising would warrant the conduct of a systematic review. An efficient way to prioritize questions could be to look at the overall risk assessment and risk management process and identify whether there are points in the process where the questions arising meet specific priority criteria (Aiassa et al., 2015), such as the need for greater precision of a parameter estimate and the clarification of uncertainty, or where extensive evidence is available that has not hitherto been formally critically appraised. An appropriate point in the risk assessment and risk management process where this could be considered is at the problem formulation step, since this step links the risk assessment and risk management process to the protection goal and effectively provides a “roadmap” for the rest of the decision-making process. The conceptual model underpinning the problem formulation step could provide an appropriate framework for determining where systematic reviews could be most valuable.

Apart from their possible application during the regulatory approval process, systematic reviews may also help in some cases to clarify possible uncertainties about GMO impacts being controversially discussed within the scientific community. The necessity for such a transparent and traceable summary is illustrated by a recent article by Hilbeck et al. (2015), discussing the diversity of scientific opinions and the problems in achieving a scientific consensus in order to conclude about GMO safety.

## Future Prospects

For systematic reviews to support GMO risk assessment and/or risk management, it is important that stakeholders involved in the risk assessment and risk management processes are aware of the purpose of systematic reviews as well as the possible limitations of non-systematic evidence synthesis approaches. At present, it is unclear whether systematic reviews would be widely supported, for example whether lack of familiarity with the methods, limited availability of resources, and a lack of primary research data would discourage those involved in risk assessment and risk management from conducting systematic reviews. A survey of the opinions of stakeholders could help to identify key facilitators of and barriers to the use of systematic reviews in supporting GMO risk assessment and risk management. Those who conduct systematic reviews will need to be aware of the appropriate methods for conducting the review whilst those coordinating the risk assessment and risk management will need to be able to identify whether a systematic review meets adequate standards of conduct. Educational resources or guidance documents to support these information requirements may need to be provided. An advantage of systematic reviews in this respect is that their highly standardized methodology is largely independent of the topic area, so guidance on how to conduct and appraise systematic reviews is already available and can be readily obtained from research institutions and organizations in health research, where the use of systematic reviews has a long pedigree.

## Author Contributions

CK and GF drafted the manuscript and all authors read, revised, and approved the final manuscript.

## Conflict of Interest Statement

The authors declare that the research was conducted in the absence of any commercial or financial relationships that could be construed as a potential conflict of interest.

## Footnotes

^1^http://www.campbellcollaboration.org/

^2^http://www.environmentalevidence.org

^3^https://www.nice.org.uk/guidance/published?type=ta

^4^https://www.nice.org.uk/news/nice-statistics
